# Coxsackievirus A16 induced neurological disorders in young gerbils which could serve as a new animal model for vaccine evaluation

**DOI:** 10.1038/srep34299

**Published:** 2016-09-26

**Authors:** Yi-Sheng Sun, Ya-jing Li, Yong Xia, Fang Xu, Wei-wei Wang, Zhang-Nv Yang, Hang-Jing Lu, Zhi-Ping Chen, Zi-Ping Miao, Wei-Feng Liang, Zhi-Yao Xu, Hong-Jun Dong, Dan-Hong Qiu, Zhi-Yong Zhu, Stijn van der Veen, Jie Qian, Bin Zhou, Ping-Ping Yao, Han-Ping Zhu

**Affiliations:** 1 Key Lab of Vaccine against Hemorrhagic Fever with Renal Syndrome, Zhejiang Provincial Center for Disease Control and Prevention, Hangzhou, China; 2 Sinovac Biotech Co., Ltd., Beijing, China; 3 The First Affiliated Hospital, Zhejiang University, Hangzhou, China; 4 Central Lab of Biomedical Research Center, Sir Run Shaw Hospital, School of Medicine, Zhejiang University, Hangzhou, Zhejiang, China; 5 Ningbo Municipal Center for Disease Control and Prevention, Ningbo, China; 6 Taizhou Municipal Center for Disease Control and Prevention, Taizhou, China; 7 Department of Microbiology and Parasitology, Collaborative Innovation Center for Diagnosis and Treatment of Infectious Diseases, School of Medicine, Zhejiang University, Hangzhou, China; 8 College of Pharmaceutical Sciences, Zhejiang University of Technology, Hangzhou, China

## Abstract

Coxsackievirus A16 (CA16) is one of the major pathogens associated with human hand, foot, and mouth disease (HFMD) in the Asia-pacific region. Although CA16 infections are generally mild, severe neurological manifestations or even death has been reported. Studies on CA16 pathogenesis and vaccine development are severely hampered because the small animal models that are currently available show major limitations. In this study, gerbils (*Meriones unguiculatus*) were investigated for their suitability as an animal model to study CA16 pathogenesis and vaccine development. Our results showed that gerbils up to the age of 21 days were fully susceptible to CA16 and all died within five days post-infection. CA16 showed a tropism towards the skeletal muscle, spinal cord and brainstem of gerbils, and severe lesions, including necrosis, were observed. In addition, an inactivated CA16 whole-virus vaccine administrated to gerbils was able to provide full protection to the gerbils against lethal doses of CA16 strains. These results demonstrate that gerbils are a suitable animal model to study CA16 infection and vaccine development.

Coxsackievirus A16 (CA16) is one of the major pathogens associated with human hand, foot, and mouth disease (HFMD) and in some countries this pathogen is the leading cause of this disease^1^,^2^. In recent years, there have been several large-scale outbreaks of HFMD in the Asia-Pacific region and therefore HFMD has become a serious public health problem in these areas^3^,^4^,^5^. Between 2008 and 2014, CA16 accounted for 22% of the HFMD cases in China, making it the second most common cause of HFMD after Enterovirus 71 (EV71)^6^,^7^. While patients infected with EV71 tend to have serious complications, patients infected with CA16 usually show mild, self-limiting disease symptoms^8^. However, some studies have reported poor clinical outcomes in patients infected with CA16, such as fatal myocarditis, pneumonia, aseptic meningitis and encephalitis^9^,^10^,^11^. Furthermore, several studies have reported high incidences of co-infections containing both EV71 and CA16^12^,^13^, while increasing numbers of clinical cases have shown that an EV71 and CA16 co-infection resulted in a worse clinical outcome^14^. Unfortunately, no treatment for HFMD is available yet. Vaccination has been suggested to be the most effective strategy to protect children from HFMD. The first inactivated EV71 vaccine has been approved by the China Food and Drug Administration (CFDA) and this vaccine showed its efficacy, safety, and immunogenicity in phase 3 clinical trials in China^15^,^16^. However, compared with EV71, less attention has been paid to CA16 and therefore research on CA16 vaccine development is still in a pre-clinical stage^14^,^17^.

Humans are the only known natural host of CA16, which complicates research on pathogenicity and vaccine development. To accelerate studies on vaccine development, a suitable CA16 animal infection model is urgently needed. Recently, a neonatal mouse model for CA16 infection was established for vaccine evaluation^18^. Using this model, passive immunization assays showed that both inactivated and virus-like particle-based vaccines provided protective immunity against CA16^14^,^19^. However, the sensitive period of the neonatal mouse model to CA16 infections was only one week, and the mouse model became less sensitive after it had reached two weeks of age^18^. This short sensitive period to CA16 infections limited the use of the mouse model in anti-viral drug development and vaccine evaluation. Active immunization assays, which required more than one week, could not be performed for an entire immunization process. Although a mouse-adapted strain of CA16 (CA16-MAV) was able to infect 2-week-old mice and cause death, it was time-consuming to acquire mouse-adapted strains from CA16 clinical isolates^20^. In addition, the considerable numbers of nucleotide variations in VP1 between clinical isolates and mouse-adapted strains indicated that mouse-adapted strains are unable to represent all the typical characters of clinical viruses^18^,^20^. *Meriones unguiculatus* (gerbils) might be a suitable alternative infection model to evaluate CA16 vaccines. This model had been used extensively to study cerebral infarction, filariasis and hemorrhagic fever with renal syndrome for years^21^,^22^,^23^. In our previous study, we showed that an EV71-infected gerbil model mimicked neurological lesion-related symptoms such as hind limb paralysis, slowness, ataxia and lethargy^24^. In the current study, we established that a clinical isolate of CA16 was highly pathogenic to gerbils. Gerbils up to 21 days of age were stably infected with CA16, which eventually resulted in dead due to neurological lesions. Virus titers were detected in the lung, liver, spleen, kidney, heart, blood, spinal cord, brain, brainstem and skeletal muscle of infected gerbils, and histological lesions were observed in the skeletal muscle, brainstem and spinal cord. The CA16-infected gerbil model was used to evaluate the protective efficacy of inactivated CA16 vaccines. An inactivated CA16 whole-virus vaccine prepared by our lab could protect gerbils from lethal challenges of CA16 with reduced virus loads and no pathological changes in different tissues. Neutralizing antibodies sustained to 12 weeks were also detected. Collectively, these results show that gerbils are a promising animal model for CA16 infection and vaccine development.

## Results

### Correlation between gerbil age and susceptibility to a CA16 infection

**Figure 1:**
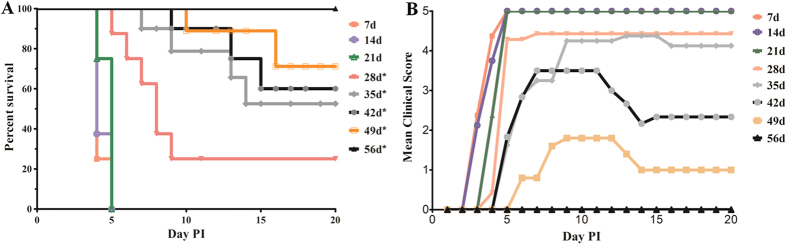
Age-related survival and severity of disease in CA16-infected gerbils. (**A**) Survival curves for groups of gerbils (n = 8–10) aged 7 to 56 days when infected with CA16-194 at a TCID_50_ of 10^5.5^. Curves were compared with the 7 days age group using the log-rank test. *Significantly different from the control group (p < 0.01). (**B**) Mean clinical scores for groups of gerbils aged 7 to 56 days when infected with CA16-194 at a TCID_50_ of 10^5.5^. One representative of two independent experiments was shown in A and B.

To investigate the correlation between the age of gerbils and their susceptibility to a CA16 infection, gerbils aged 7 to 56 days were inoculated via the intra-peritoneal (IP) route using clinical isolate CA16-194 at a 50% tissue culture infective dose (TCID_50_) of 10^5.5^ per gerbil. Gerbils were monitored for survival and severity of clinical symptoms for 20 days (Fig. 1A). Gerbils up to 21 days of age all died within five days post-infection (Fig. 1A), which was the result of a rapid onset of clinical symptoms at three to four days post-infection (Fig. 1B). The percentage of survival increased with the age of infection and similarly, the clinical symptoms were also reduced in older gerbils. Finally, gerbils at 56 days of age did not show any clinical symptoms after infection with CA16-194 and hence, all gerbils survived. These results indicated that gerbils aged 21 days or younger were fully susceptible to a CA16 infection and owing to the long sensitive period, 21-day-old gerbils were selected for further analysis of their suitability as an animal model.

### Correlation between CA16 dose and severity of disease in gerbils

**Figure 2:**
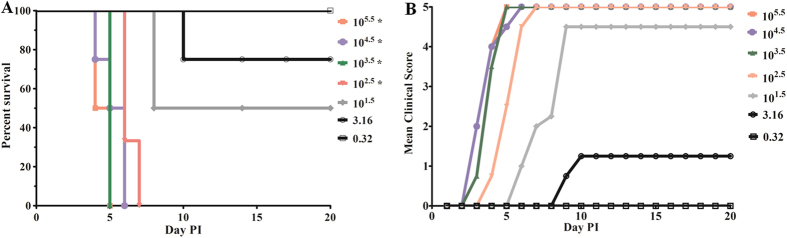
Dose-dependent survival and severity of disease in CA16-infected gerbils. (**A**) Survival curves for groups of gerbils (n = 8–10) aged 21 days when infected with CA16-194 at a TCID_50_ of 0.3 to 10^5.5^. Curves were compared with the 0.3 TCID_50_ group using the log-rank test. *Significantly different from the control group (p < 0.01). (**B**) Mean clinical scores for groups of gerbils aged 21 days when infected with CA16-194 at a TCID_50_ of 0.3 to 10^5.5^. One representative of two independent experiments was shown in A and B.

In order to further evaluate 21-day-old gerbils as a suitable CA16 infection model, gerbils were infected with a TCID_50_ ranging between 10^5.5^ and 0.32 using CA16-194. Gerbils infected with higher doses, TCID_50_ of 10^2.5^ and above, all died within four to seven days after infection (Fig. 2A), while clinical symptoms appeared at three to four days post-infection (Fig. 2B). At lower doses of CA16-194, onset of symptoms was retarded and survival of the gerbils was increased. Finally, at a TCID_50_ of 0.32, all gerbils survived and remained fully healthy. Based on these results, the 50% lethal dose (LD_50_) was calculated to be 3.16 × 10^1.0^ TCID_50_. These results showed that the disease severity and clinical outcome of CA16-infected gerbils were correlated with the dose of infection.

### Dissemination and replication of CA16 in gerbil tissues

**Figure 3:**
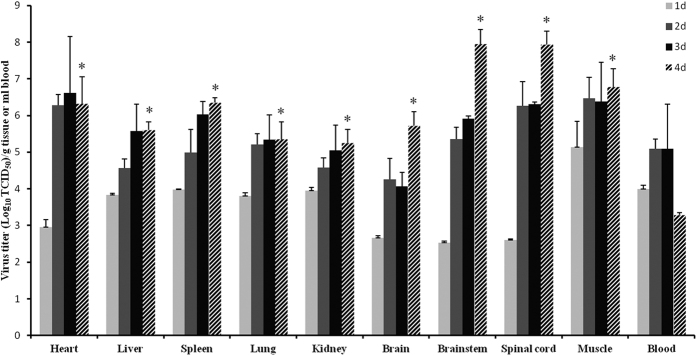
Virus dissemination and replication in different tissues of CA16-infected gerbils. 21-day-old gerbils were inoculated with CA16-194 at 100 × LD_50_. The graph shows the virus loads in the heart, liver, spleen, lung, kidney, brain, brainstem, spinal cord, muscle and blood from CA16-infected gerbils at 1, 2, 3 and 4 days post infection. Virus loads were assessed by real-time RT-PCR and compared with standard curves obtained from 10-fold serial dilutions of CA16-194. Results represent the mean ± standard error of the virus titer (log_10_ TCID_50_) per gram of tissue or per milliliter of blood from two independent experiments using three technical replicates each. ^*^Significantly different from the day 1 group in each tissue (p < 0.01).

To investigate dissemination of CA16 in infected gerbils and replication of the virus in different organs and tissues, gerbils at the age of 21 days were infected with a TCID_50_ of 10^5.5^ and virus loads were monitored in the heart, liver, spleen, lung, kidney, brain, brainstem, spinal cord, muscle and blood at one to four days post-infection. Virus replication was observed in all of the tissues investigated (Fig. 3). At one day post-infection, the highest virus load was detected in muscle tissues, with a TCID_50_ of 10^5.1^ per gram (Fig. 3). Virus loads generally increased until two to three days post-infection, after which a plateau was reached for most of the tissues. Interestingly, in the nervous tissues (brain, brainstem and spinal cord), virus loads reached an initial plateau at two days post-infection, but at four days post-infection the virus loads showed again an increase. As a result, the highest virus titers were recorded for the brainstem and spinal cord, with a TCID_50_ of 10^7.9^ per gram. The high virus loads detected in the gerbil muscle, brainstem and spinal cord indicated that these tissues were major targets during CA16 infection, and damage to their structural integrity might therefore play an important role in the onset of typical disease symptoms such as wasting and hind-limb paralysis.

### Pathology of CA16-infected gerbil tissues

**Figure 4:**
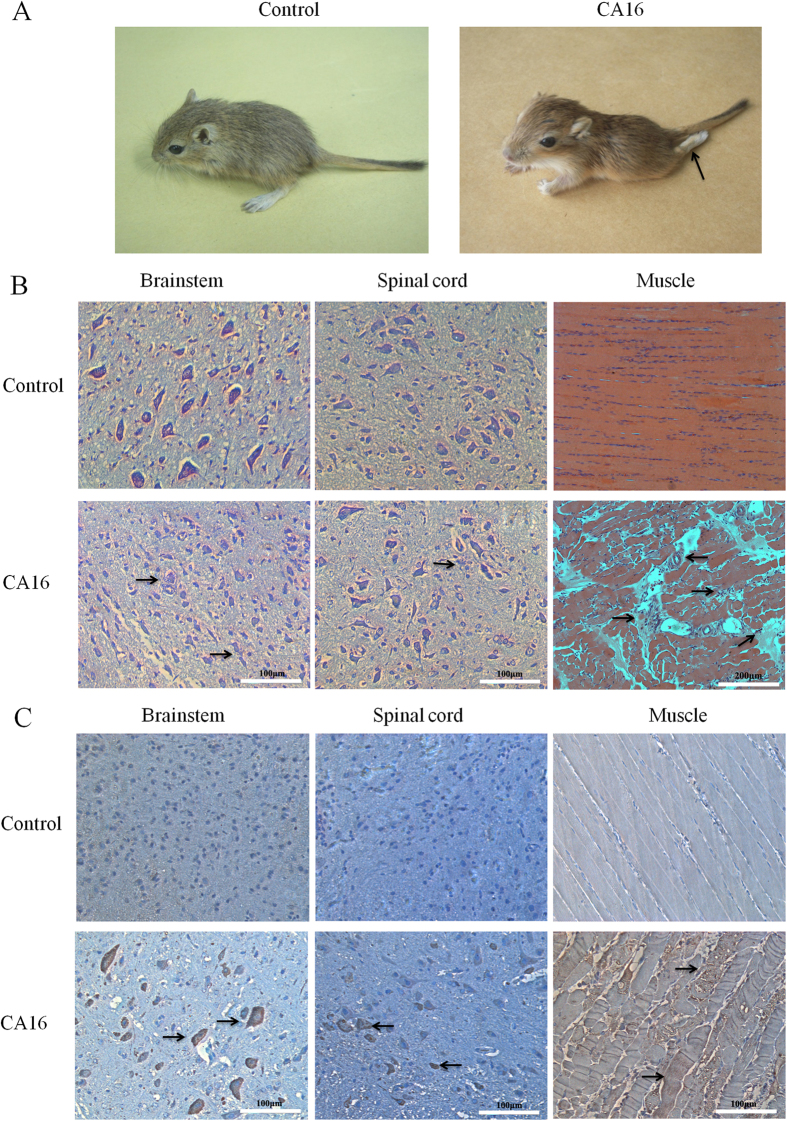
Pathology of CA16-infected gerbil tissues. Gerbils aged 21 days were challenged with CA16-194 using a TCID_50_ of 10^5.5^. (**A**) Representative images of control and CA16-infected gerbils four days post infection. The arrow indicates hind-limb paralysis observed in the CA16-infected gerbils. (**B**) Representative images of haematoxylin and eosin or Nissl stained brainstem, spinal cord and muscle tissues harvested from CA16-infected and control gerbils four days post infection. The arrows indicate focal shrunken neurons in the infected brainstem, swollen neurons and neuronophagia in the infected spinal cord, and inflammatory cell infiltration, severe necrotizing myositis, degeneration and swelling of skeletal muscle fibers in the infected muscle. (**C**) Representative images of brainstem, spinal cord and muscle tissues harvested from CA16-infected and control gerbils four days post infection. Presence of viral antigens (arrows) was visualized by incubation with peroxidase staining DAB followed by counterstaining with haematoxylin.

To investigate the pathological effects of CA16 on infected gerbil tissues, 21-day-old gerbils were infected with CA16-194 at a TCID_50_ of 10^5.5^. At four days post-infection, the brainstem, spinal cord and muscle tissues were harvested and analyzed. Gerbils at this stage all showed typical clinical symptoms such as wasting and hind-limb paralysis (Fig. 4A). The brainstem and spinal cord were the most severely affected organs of the central nervous system (CNS) in the infected gerbils. In the infected brainstem, focal shrinking neurons were detected (Fig. 4B). In the spinal cord, neurons were found to be swollen and neuronophagia was detected (Fig. 4B). Finally, inflammatory cell infiltration and severe necrotizing myositis were observed in infected muscle tissues. Degeneration and swelling of skeletal muscle fibers were also detected, which resulted in muscle cell dead (Fig. 4B). In addition, widespread dissemination of the CA16 antigen was observed and corresponded with lesions present in the brainstem, spinal cord and muscle tissues (Fig. 4C). These results indicated that CA16-194 showed a strong tropism towards central nervous and skeletal muscle tissues.

### Protective efficacy of a CA16 vaccine in gerbils

**Figure 5:**
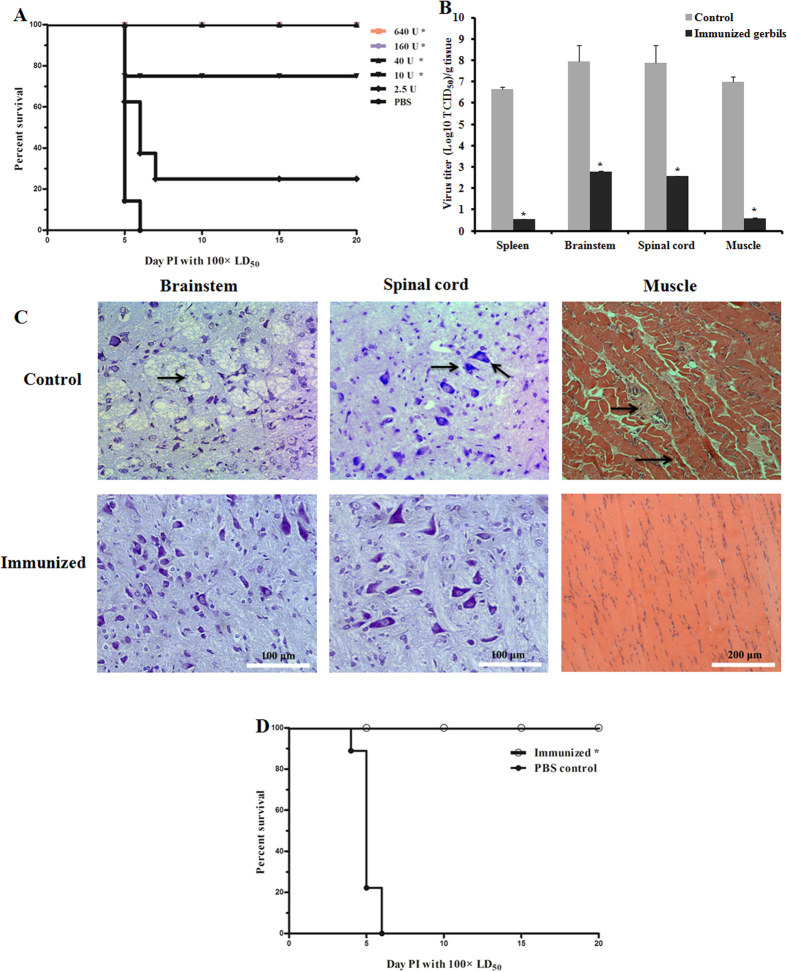
Protective efficacy against lethal CA16 challenges in immunized gerbils. Gerbils were immunized with a CA16 vaccine at the age of 7 and 14 days and challenged at the age of 21 days with CA16 virus using a 100 × LD_50_. (**A**) Survival curves of groups of gerbils (n = 8) were immunized with the CA16 vaccine using a dose of 2.5, 10, 40, 160, 640 U or PBS as a negative control, then challenged with strain CA16-194. Curves were compared with the PBS control group using the log-rank test. Representative results from two similar experiments are shown. ^*^Significantly different from the control group (p < 0.01). (**B**) Viral loads in the spleen, brainstem, spinal cord and muscle tissue of gerbils four days post infection. Gerbils were immunized with the CA16 vaccine using a dose of 160 U or a PBS control, then challenged with strain CA16-194. Virus loads were assessed by real-time RT-PCR and compared with standard curves obtained from 10-fold serial dilutions of CA16-194. Results represent the mean ± standard error (n = 3 each) of the virus titer (log_10_ TCID_50_) per gram of tissue. ^*^Significantly different from the control group (p < 0.01). (**C**) Histology examinations of brainstem, spinal cord (Nissl stained) and muscle tissues (haematoxylin and eosin stained) harvested from CA16 vaccine-immunized and control gerbils 4 dpi. Gerbils were immunized with the CA16 vaccine using a dose of 160 U or a PBS control, then challenged with strain CA16-194. The arrows indicated shrinking neurons and sieve-like changes in brainstem, neuronophagia in the spinal cord, degeneration of skeletal muscle fibers and inflammatory cell infiltration in muscle of PBS controls. (**D**) Survival curves of groups of gerbils (n = 8) were immunized with the CA16 vaccine using a dose of 160 U or PBS as a negative control, and then challenged with CA16-196 using a 100 × LD_50_. Curves were compared with the PBS control group using the log-rank test. ^*^Significantly different from the control group (p < 0.01).

To investigate gerbils as a model to evaluate vaccine efficacy, the protective efficacy of purified formalin-inactivated CA16 vaccines was analyzed. Groups of 7-day-old gerbils (n = 8 for each group) were immunized with the purified inactivated CA16-193 vaccine at a dose ranging from 2.5 to 640 units (U), or a control (PBS). Vaccination was repeated when gerbils were 14 days of age, and finally at 21 days of age gerbils were challenged with CA16-194 using a 100 × LD_50_. All gerbils of the control group rapidly developed limb paralysis during the course of infection and finally died at five to six days post-infection. In contrast, from the group of gerbils vaccinated with the lowest dose (2.5 U), 25% (2 of 8) survived the lethal challenge (Fig. 5A). Furthermore, 75% of the gerbils vaccinated at a dose of 10 U were protected and at higher vaccine doses (≥40 U), all gerbils survived (Fig. 5A). Subsequently, viral loads were analyzed in the tissues of vaccinated and control animals four days after a lethal CA16-194 challenge. Viral loads were significantly reduced in the spleen, brainstem, spinal cord and muscle tissues of vaccinated gerbils compared with control animals (Fig. 5B). No pathological changes of the brainstem, spinal cord and muscle were detected in vaccinated gerbils four days after a lethal challenge of CA16-194. However, in the PBS control group, shrinking neurons and sieve-like changes in the brainstem, neuronophagia in the spinal cord, and degeneration of skeletal muscle fibers and inflammatory cell infiltration in muscle tissues were found (Fig. 5C). The cross-protective capacity of the CA16-193 vaccine was also evaluated. Vaccinated gerbils were challenged by another clinical strain, CA16-196. Gerbils inoculated with the CA16-193 vaccine at a dose of 160 U all survived, while gerbils in the control group all died (Fig. 5D). These results indicated that the vaccine-induced immune response was sufficient to prevent viral replication and pathological changes in the challenged gerbils, which resulted in survival of gerbils from an otherwise lethal CA16 challenge.

### Neutralizing antibody responses in immunized gerbils

To investigate whether gerbils were able to elicit neutralizing antibody responses after vaccination, 21-day-old gerbils were immunized with a dose ranging from 2.5 to 640 U of the CA16 vaccine (n = 8 each group). A booster dose was given after two or four weeks and neutralizing antibody responses were subsequently determined after 1, 2, 3, 5, 8 and 12 weeks. In all instances, neutralizing antibodies peaked in the first week after the booster dose and subsequently waned (Table 1). Protective neutralizing antibody levels are estimated at a titer above 1:8^25^. Gerbils that received a booster vaccine at a dose of 160 and 640 U after a four-week interval maintained sufficiently high neutralizing antibody titers to provide protection until 12 weeks post-infection. At lower doses, no protective levels of neutralizing antibodies were reached. Gerbils receiving a booster vaccine after a two-week interval using a dose of 160 and 640 U maintained protective neutralizing antibody levels for five and eight weeks, respectively, while gerbils receiving a dose of 10 or 40 U only maintained protective levels for one week. These results indicated that immunization of gerbils with two sufficiently high doses of an inactivated CA16 whole-virus vaccine resulted in protective neutralizing antibody titers.

## Discussion

Gerbils (*M. unguiculatus*) are rodents that belong to the subfamily *Gerbillinae* and are slightly bigger than mice. They have many advantages as animal model of human disease. In our previous studies, we demonstrated that young gerbils were susceptible to EV71 infections and EV71 showed a high level of pathogenicity^24^. Gerbils infected with EV71 could mimic the pathology observed in humans. Both EV71 and CA16 belong to the enterovirus genus and cause similar clinical symptoms^18^. However, no study has demonstrated thus far whether CA16 is able to infect gerbils as well. In this study, CA16-194, a strain of CA16 isolated from a swab sample of a 12-month-old boy with HFMD, was used to study infection of gerbils. The VP1 gene of this strain was sequenced and the strain was classified into the B1 subtype, which in recent years has been the major subtype of CA16 in mainland China^26^. Our studies showed that this clinical CA16 isolate was able to induce neurological lesion-related symptoms in gerbils, which resembled the symptoms observed in human patients. Gerbils aged 21 days and lower all suffered from clinical symptoms and died within 5 days after infection by the IP route. Analysis of virus loads in nine tissues from infected gerbils showed that the muscle, brainstem and spinal cord contained the highest virus titer. Severe necrosis in skeletal muscle, spinal cord and brainstem were also observed by pathological examination and immunohistochemistry staining, indicating that these were indeed the major target tissues for virus replication. This work indicated that CA16 exhibited neurotropism in gerbils and gerbils might therefore be a suitable animal model to study CA16 infection. In humans, the natural route of infection by CA16 is via the oral (OL) route. Therefore, we also investigated the susceptibility of gerbils to infection via the OL route. However, only three out of eight gerbils that were OL inoculated with CA16 at a dose of 10^5.5^ TCID_50_ showed disease symptoms and the onset time was delayed to 6–8 dpi (see Supplementary Fig. S1). Compared with IP administration, gerbils were less sensitive to infection via the OL route, which was also found in the EV71-infected gerbil model in our previous work^27^. The IP route might therefore be a better choice than the OL route for CA16 inoculation of gerbils.

Using a suitable animal model is essential to obtain a better understanding of pathogen transmission and distribution, the mechanisms of virus infection, the incidence of pathological lesions, host immune responses and vaccine efficacy. Previously, rhesus monkeys were reported as a useful model for EV71 infection, however, this animal model had never been evaluated for its suitability to study CA16 infections. Compared with large animals, small animal models are more convenient and cost-effective to study the mechanism of viral pathogenesis. However, only the neonatal mouse model has been established as a CA16-infection model^17^,^18^. More suitable animal models should therefore be developed. We observed that gerbils aged 7 to 21 days all died when infected with clinical CA16 isolates. Even 35-day-old gerbils were still efficiently infected with CA16, resulting in severe symptoms and nearly 50% mortality. Infection of 7-day-old gerbils resulted in disease symptoms at four days post-infection, which is earlier than observed in neonatal mice. Infection with 2.3 × 10^5.0^ TCID_50_ of a mouse-adapted virus (MAV) induced symptoms in 14-day-old mice, while 21-day-old gerbils suffer from severe symptoms and 100% mortality after infection with a dose as low as 10^2.5^ TCID_50_ using a clinical CA16 isolate. Although adaptation of CA16 to mice might impact virus pathogenicity^20^, it appears that gerbils are more sensitive to CA16 than neonatal mice. Also, in the neonatal mouse model, CA16 is more virulent than EV71 with the LD_50_ of CA16 approximately 100-fold lower than that of EV71^18^,^28^. In the gerbil model, the LD_50_ of EV71 and CA16 were 1 × 10^2.68^ TCID_50_ and 3.16 × 10^1.0^ TCID_50_, respectively, indicating that gerbils were more susceptible to CA16 than to EV71.

Severe HFMD in patients infected with CA16 is usually accompanied by nervous system damage or neurological complications that are difficult to be cured. Increasing evidence shows that a CA16 infection is associated with damage to muscle and brain tissues^29^,^30^,^31^. Furthermore, clinical CA16 strains with or without neurological virulence are able to induce neural and muscle cell apoptosis *in vitro*^32^. Therefore, it appears that CA16 displays a strong tendency for neurotropism and myotropism. However, in the neonatal mouse model, severe necrosis and presence of CA16 antigens are only observed in skeletal muscle fibers. Neither pathological changes nor CA16 antigens are found in the neurocytes of the brain or the spinal cord, indicating that CA16 does not display significant neurotropism in the neonatal mouse model^17^,^18^. Also, intensive-care patients infected with the CA16 virus usually have serious nervous system diseases, but again, the neonatal mouse model does not reflect these clinical symptoms very well. In contrast, in the gerbil model, CA16 antigens were detected not only in the hind-limb skeletal muscles, but also in the spinal cord and brainstem. Furthermore, both CNS damage (neuronophagia in the spinal cord and shrinking neurons in the brainstem) and muscle damage (necrosis in the hind-limb muscle) were observed in gerbils, which was consistent with clinical symptoms observed in patients. Therefore, it appeared that gerbils were a good animal model to study nervous system damage during a CA16 infection. In addition, neonatal mice are so small that IC or IP inoculation of the brain or abdominal cavity is very tricky. Leakage often occurs during the inoculation procedure, which results in an inaccurate immunization procedure. Since gerbils are slightly bigger than mice at the same age, the inoculation procedure is more convenient. Also, mice older than 7 days are not susceptible to a CA16 infection, while 21-day-old gerbils are still fully susceptible. Therefore, gerbils offer major advantages for studying the pathogenic mechanism as well as evaluating the immune protection of vaccine candidates.

Although it has been shown recently by *in vitro* assays that natural compounds such as corilagin or luteolin are able to inhibit CA16 infection^33^,^34^, effective anti-viral drugs for HFMD remain unavailable. Vaccination is still considered to be one of the most important strategies to prevent this disease. However, before entering into clinical trials, protective efficacy and safety of vaccines have to be tested in animal models. Although the neonatal mouse model has been established for evaluating the protective efficacy of CA16 candidate vaccines, immunizing 1-day-old pups is not only technically difficult but also not suitable for evaluation of CA16 vaccines. Mao *et al.* tested maternal immunization of mice instead. After analyzing the clinical symptoms and survival rates of infected neonatal mice, it appeared that maternal immunization only resulted in passive immunity transferred from the mother mouse to its offspring^18^. In addition, the procedure of passive immunization suffered from external factors, including the inability of mice to get pregnant or low numbers of newborn mice. Cai *et al.* investigated a different approach. They developed a CA16 mouse-adapted strain, which prolonged the sensitive period of mice to 2 weeks, and used it to evaluate active immunization of a vaccine candidate^20^. However, using a mouse-adapted strain to evaluate the protective effects of a vaccine raises the question whether a mouse-adapted strain is able to represent all the specific characteristics of clinical strains. Several studies have shown that the adaptation process resulted in mutations in conserved amino acids of the VP1-VP4 or 2C genes, which had a significant impact on the pathogenesis of clinical strains^35^,^36^,^37^,^38^. Sequence analysis of VP1 in the mouse-adapted CA16 strain showed that its amino acid sequence was only 98% identical to that of the parent strain and several key amino acids were mutated. Thus far, no small animal model has been developed to evaluate the protective effects of active immunization against challenges of clinical strains. In the gerbil model, the sensitive period to a CA16 infection was much longer compared with mice. Gerbils up to the age of 21 days were fully susceptible to CA16 and they were suitable to evaluate the protective effects of vaccine candidates. In our hands, an effective protocol was to immunize 7-day-old gerbils via the IP route, provide a booster dose a week later, and challenge gerbils at 21 days of age using CA16 at a 100 × LD_50_. Gerbils immunized with inactivated CA16-193 whole-virus vaccines were protected from lethal challenges of clinical strains CA16-194 and CA16-196. Furthermore, susceptibility to clinical CA16 strains was still observed in gerbils up to the age of 49 days, allowing for adaptation of protocols that required different immunization schedules. Therefore, gerbils are a convenient and reproducible animal model to study vaccine efficacy against CA16, and it offers major advantages over the neonatal mouse model.

Previous protection experiments using a CA16 vaccine have demonstrated that mice immunized with two doses of the vaccine were fully protected from virus challenges, indicating that two doses of a formalin-inactivated CA16 vaccine should be sufficient to provide protection. After immunization, the challenged mice were monitored for 18 days, which represented a short-time protective effect against CA16^20^. However, a safe and efficacious vaccine should also provide long-term protections. Virus-neutralizing antibodies are the key to protect against CA16 challenges and a titer above 1:8 in neutralization assays is considered as sufficient for protective immunity^25^. Although CA16-193 and CA16-194 were purified from the same patient, antigens from strain CA16-193 were more stable than those from CA16-194 and therefore we selected strain CA16-193 to produce the inactivated CA16 whole-virus vaccines (see Supplementary Fig. S3). In order to evaluate the long-term protective efficacy of our vaccine, two doses of the vaccine were administered to the gerbils at a 2-week or 4-week interval. Neutralizing antibodies against CA16 were detected in the serum up to 12 weeks after the second dose was administered. However, persistent protective antibody levels were only provided by high vaccine doses (160 U and above). At these doses, the 4-week immunization interval group induced higher antibody levels and longer protection than the 2-week interval group. Apparently, a 2-week interval for the two doses is too short for the gerbils to trigger their cellular immune system and activate the corresponding signaling pathways to effectively protect gerbils from viral challenges for a longer period. In phase 3 clinical trials of an EV71 vaccine in China, vaccines were also administered to children at a four-week interval^16^, which was consistent with the observations from our immunization procedures. Interestingly, when gerbils were immunized with a low dose of the CA16 vaccine (40 or 10 U), neutralizing antibody responses were slightly better in the 2-week interval group compared with the 4-week interval group. However, at these lower doses, persistent immune responses were not ideal and neutralizing antibody levels waned rapidly. Our study demonstrated that the interval between the priming and booster immunization significantly influenced the effectiveness of our vaccine. An interval of 4 weeks between the two immunizations of the CA16 vaccine, and a dose of 160 U and above are therefore considered optimal.

In recent years, several studies have reported on the co-circulation and co-infection of EV71 and CA16^39^,^40^, but until now, co-infections appear not to be associated with more severe clinical symptoms. However, several CA16 strains have been detected in which the non-structural gene regions and the 3′-untranslated regions show evidence of recombination with EV71^41^,^42^. Recombination between these two viruses may alter their pathogenicity and transmission pattern, which can pose a serious threat. Although an EV71 vaccine, which provides high efficacy, satisfactory safety, and sustained immunogenicity, has been approved by the CFDA in December 2015^15^, an EV71-CA16 combined vaccine had not been commercialized. Development of several bivalent vaccines was ongoing, but they were all still in a pre-clinical stage^43^,^44^,^45^. Using these vaccines, little cross-activity between the EV71 and CA16-induced neutralizing antibodies was detected. More importantly, only protective effects of passive immunization were evaluated in these bivalent vaccines, while the efficacy of active immunization for both EV71 and CA16 should be established. Due to the incidence of co-circulation and co-infection of CA16 and EV71, it is essential to develop a multivalent vaccine or at least combine an EV71 and CA16 vaccine and evaluate their immunogenicity and safety. In our previous study, we established young gerbils as a suitable disease model for EV71 infection. Young gerbils infected with EV71 have a long sensitive period and develop severe pulmonary lesions and neurological lesion-related symptoms^24^. In the current study, we report that the CA16-infected gerbil model mimics neurological lesion-related symptoms and necrosis in the spinal cord and brainstem. These results indicate that gerbils may be an ideal animal model to study co-infections of EV71 and CA16. Gerbils at 21 days of age are fully susceptible to both EV71 and CA16 and the sensitive period is sufficient to perform active immunization experiments to evaluate an EV71-CA16 combined vaccine.

In conclusion, our results showed that 21-day-old gerbils infected with CA16 developed neurological lesion-related symptoms and severe necrosis in the skeletal muscle, spinal cord and brainstem, eventually resulting in dead. Gerbils immunized with the inactivated CA16 whole-virus vaccines produced by our lab were fully protected against lethal challenge of clinical strains CA16-194 and CA16-196. Therefore, gerbils are a suitable animal model to study CA16 infection and to evaluate the immunogenicity of a CA16 vaccine following an active immunization protocol.

## Methods

### Ethical statement

All animal experimental protocols were approved by the Ethics Committee of the Zhejiang Provincial Center for Disease Control and Prevention in China. The methods were carried out in accordance with the guidelines of Administration of Affairs Concerning Experimental Animals of the People’s Republic of China and principles of the Declaration of Helsinki. All human experimental protocols were approved by both the Ethics Committee of the Zhejiang Provincial Center for Disease Control and Prevention and the Hangzhou Sixth People’s Hospital in Hangzhou, China. All the methods were carried out in accordance with the principles of the Declaration of Helsinki. Written informed consents for the use of their clinical samples were obtained from all subjects (the legal guardians of the patients and contacts).

### Viral isolates and growth conditions

CA16 clinical strains CA16-193 (Genbank ID: KU854873) and CA16-194 (Genbank ID: KX056216), both belonging to genotype B1, were isolated from swab samples of a twelve-months-old boy with HFMD in the Hangzhou Sixth People’s Hospital in 2008. CA16-196 (Genbank ID: KX580041) was grouped into genotype B1b and isolated from blister fluid of a 37-month-old boy at the same hospital in 2008. The viral samples were diluted in Eagle’s minimum essential medium (MEM) supplemented with 100 units/ml of penicillin and 100 μg/ml of streptomycin prior to infection of Vero cells. Viruses were continuously passaged with Vero cells until a cytopathogenic effect (CPE) was observed. Throughout this study, strains CA16-193 and CA16-194 were used at the 5th and 3th passage, respectively. The stock of virus was grown in Vero cells and the 50% tissue culture infective dose (TCID_50_) was determined using standard methods described previously^24^. Vero cells were grown in MEM supplemented with 10% fetal bovine serum at 37 °C.

### Gerbil infection experiments

Healthy gerbils were obtained from the Animal Center of the Zhejiang Academy of Medical Sciences, Hangzhou, China. Gerbils were housed under specific pathogen-free conditions. To establish the correlation between age and susceptibility to infection, gerbils were inoculated at the age of 7, 14, 21, 28, 35, 42, 49 and 56 days (n = 8 to 10 per age group) by the intra-peritoneal (IP) route using strain CA16-194 at a TCID_50_ of 10^5.5^. The animals were observed twice daily for clinical signs, weight changes and mortality for 20 days. The grade of clinical disease was scored as follows: 0, healthy; 1, ruffled hair, hunchbacked or reduced mobility; 2, limb weakness; 3, paralysis in one limb; 4, paralysis in both limbs or deep lethargy; 5, death. To establish the correlation between dose and susceptibility to infection, 21-day-old gerbils were inoculated with CA16-194 or CA16-196 at a TCID_50_ of 0.3, 3.16, 10^1.5^, 10^2.5^, 10^3.5^, 10^4.5^ and 10^5.5^. The LD_50_ of strains CA16-194 and CA16-196 in 21-day-old gerbils was calculated by the Reed-Muench method as described previously^46^. Again, the grade of clinical disease was monitored and recorded twice daily after infection. Control animals were inoculated with PBS.

### Preparation of inactivated CA16 whole-virus vaccines

Virus CA16-193 was grown in Vero cells in MEM medium until CPE was observed and supernatant was subsequently harvested by two freeze-thaw cycles and centrifugation at 2000 g (Beckman) for 20 min. The supernatant was mixed with diluted formalin (Sigma; 1:2500) and incubated at 37 °C for 3–4 days. After inactivation, the suspension was centrifuged at 5000 g (Beckman, 25R) for 30 min and the supernatant was concentrated using a 100 K ultrafiltration membrane (Omega, USA) to 1/20 of the original volume. The concentrated suspension was loaded onto a Sepharose 6 Fast Flow column (GE Healthcare, USA) for further purification. Column fractions containing the virus were pooled and the completeness of virus inactivation was verified by the absence of CPE during three blind serial passages in Vero cells. The concentration of the purified inactivated virus suspension was tested by ELISA. Briefly, 100 μl of two-fold serial dilutions of the virus suspension was coated to a 96-well microtiter plate (Corning) and incubated at 37 °C overnight. Polyclonal CA16 rabbit antiserum (1:2000) was added to the coated plate and the plate was incubated at 37 °C for 60 min. The viral antigens were detected by horseradish peroxidase-conjugated anti-rabbit antibodies (1:5000; Sigma–Aldrich). Optical densities (ODs) were read in a microtiter plate reader (Bio-Rad) at 450 nm and values over 0.125 were considered positive. The maximum dilution of the virus suspension that showed a positive signal was 1:400 and the concentration of this solution was therefore defined as 4000 U/ml to facilitate further calculations. The dosage of the virus suspension in the final vaccine formulation was adjusted with PBS and aluminum hydroxide (National Vaccine and Serum Institute, China) was added in a final concentration of 0.6 mg/ml. The vaccine formulations were shaken for 1 h and stored at 4 °C until use.

### Immunization and neutralizing antibody responses

Gerbils aged 21 days were divided into two times five groups (8–10 gerbils per group) and IP immunized with two doses of the formaldehyde-inactivated CA16 virus vaccines at an interval of two weeks or four weeks. For each time interval, five groups of gerbils were immunized with a dose of 640, 160, 40, 10, 2.5 U or a negative control and the same dose was repeated at an interval of two or four weeks. Blood samples were collected from the orbit of the gerbils 1, 2, 3, 5, 8 and 12 weeks after the second immunization. The serum fraction of the blood was harvested and inactivated at 56 °C for 30 min. 25 μl of serial two-fold dilutions of serum was mixed with 25 μl of CA16-194 at a 100 × TCID_50_ and incubated at 37 °C for 2 h to neutralize infectious viruses. The mixtures were subsequently transferred to 96-well plates containing a >90% confluent monolayer of Vero cells in MEM supplemented with 5% FBS. After incubation for 5 days at 37 °C until CPE was observed in control wells containing a 100 × TCID_50_ of active virus, the neutralizing antibody titer was regarded as the highest dilution of serum that completely inhibited virus growth (inhibiting CPE). The experiment was performed in duplicate and the average neutralization titer was reported.

### Immunization and viral challenges

Gerbils at seven days of age were randomly divided into different experimental or control groups (n = 8–10 per group). Each group of gerbils was immunized IP with a different dose of the vaccine (640, 160, 40, 10, 2.5 U) or a control. After a week (14 days of age), the same vaccine was repeated as a booster vaccine and another week later (21 days of age) the gerbils were challenged with viral strains CA16-194 or CA16-196 at a 100 × LD_50_. All gerbils were monitored daily for disease symptoms until 20 days after challenge. The LD_50_ for CA16-196 in gerbils was determined at a TCID_50_ of 10^1.5^ (see Supplementary Fig. S2).

### Quantitative detection of CA16 RNA by RT-PCR

21-day-old gerbils were inoculated with CA16-194 (100 × LD_50_). Gerbil heart, liver, spleen, lung, kidney, brain, brainstem and spinal cord tissues and blood were aseptically collected and 25 mg tissue or 25 μl blood was homogenized in lysis buffer. The lysates were subjected to three freeze-thaw cycles and subsequently centrifuged at 12,000 g for 10 min. The supernatants were harvested and viral RNA was extracted using the RNeasy extraction kit (Qiagen, USA) according to the manufacturer’s protocols. RNA was eluted in 30 μl nuclease-free water and stored at −80 °C. Viral loads in the various tissues were evaluated by CA16 TaqMan RT-PCR. Reactions were performed with a one-step RT-PCR kit (Liveriver, ZJ Bio-Tech Co., Ltd. Shanghai). cDNA was synthesized from RNA by reverse transcription for 30 min at 42 °C and subsequently amplified for 40 cycles at 95 °C for 5 s and 60 °C for 30 s. Each assay was performed in triplicate. As described previously^24^, the standard curve was developed with serial 10-fold dilutions of virus stock (strain CA16-194 with 10^7.0^ TCID_50_/ml). RNA from the negative-control group was evaluated simultaneously in each TaqMan RT-PCR.

### Histology examination

Gerbils at the age of 21 days were infected with strain CA16-194 at a TCID_50_ of 10^5.5^ and a 100 × LD_50_. After four days infection, the gerbils were sacrificed and subjected to histopathologic and immunohistochemical examination. Tissue samples (skeletal muscle, spinal cord, and brainstem) of the gerbils were obtained immediately after anesthetization, fixed in 10% neutral buffered formalin for two days, dehydrated in ascending concentrations of ethanol and embedded in paraffin. For each tissue specimen, several sections were sliced, mounted on poly-L-lysine-coated slides and stained with haematoxylin and eosin or with Nissl before histopathologic examination. For immunohistochemical testing, tissue sections were dewaxed, dehydrated, and incubated with 0.3% H_2_O_2_ in PBS for inhibiting endogenous peroxidase activity. Polyclonal rabbit anti-CA16 antibodies (1:100 dilution), produced by immunization of rabbits using whole inactivated CA16 virus, were added and samples were incubated for 1 h at room temperature. A peroxidase-conjugated anti-rabbit antibody (1:200 dilution; Cell Signaling Technology, Beverly) was added for 30 min at room temperature. Viral antigens in the tissue sections were visualized by incubation with the peroxidase stain DAB (Dako, Glostrup, Denmark), which was followed by counterstaining with Mayer’s haematoxylin (Merck, Darmstadt, Germany).

### Statistical Analysis

The clinical scores and virus loads were analyzed by the nonparametric one-way analysis of variance (ANOVA) or two-tailed student’s t-test. Survival curves were evaluated by the Mantel-Cox log-rank test and plotted by GraphPad Prism, version 5.0 (GraphPad 4 Software, San Diego, CA). Differences were considered significant at p < 0.01.

## Additional Information

**How to cite this article**: Sun, Y.-S. *et al.* Coxsackievirus A16 induced neurological disorders in young gerbils which could serve as a new animal model for vaccine evaluation. *Sci. Rep.* **6**, 34299; doi: 10.1038/srep34299 (2016).

## Supplementary Material

Supplementary Information

## Figures and Tables

**Table 1:** Gerbil neutralizing antibody responses elicited by a CA16 vaccine.

Immunization interval	Vaccine dose (U)	Neutralizing antibodies (1/GMT)					
		1w	2w	3w	5w	8w	12w
2 weeks	640	45.99	25.01	20.64	30.37	8.7	6.07
	160	20.06	11.00	11.23	9.77	3.23	5.15
	40	19.65	4.75	1.41	0	0	0
	10	19.41	1.28	1.29	0	0	0
	2.5	2.49	0	0	0	0	0
4 weeks	640	120.58	97.60	84.33	52.46	30.69	24.69
	160	179.55	113.00	26.63	30.21	30.88	13.51
	40	5.26	1.04	0	0	0	0
	10	1.86	0	0	0	0	0
	2.5	0	0	0	0	0	0

GMT: geometric mean titers.
