# Supplementary material for: Coxsackievirus A16 induced neurological disorders in young gerbils which could serve as a new animal model for vaccine evaluation
Source: Sci Rep. 2016 Sep 26;6:34299. doi: 10.1038/srep34299 (PMC5035925; doi:10.1038/srep34299)
Supplement: Supplementary Information [file srep34299-s1.doc]

**Coxsackievirus A16 induced neurological disorders in young gerbils which could serve as a new animal model for vaccine evaluation**

Yi-Sheng Sun^1,*^, Ya-jing Li^2,*^, Yong Xia^1,*^, Fang Xu^1^, Wei-wei Wang^2^, Zhang-Nv Yang^1^, Hang-Jing Lu^1^, Zhi-Ping Chen^1^, Zi-Ping Miao^1^, Wei-Feng Liang^3^, Zhi-Yao Xu^4^, Hong-Jun Dong^5^, Dan-Hong Qiu ^6^, Zhi-Yong Zhu^1^, Stijn van der Veen^7^, Jie Qian^8^, Bin Zhou^8^, Ping-Ping Yao^1^, Han-Ping Zhu^1^

^1^Key Lab of Vaccine against Hemorrhagic Fever with Renal Syndrome, Zhejiang Province Center for Disease Prevention and Control, Hangzhou, China

^2^Sinovac Biotech Co., Ltd., Beijing, China

^3^The First Affiliated Hospital, Zhejiang University, Hangzhou, China

^4^Central Lab of Biomedical Research Center, Sir Run Shaw Hospital, School of Medicine, Zhejiang University, Hangzhou, Zhejiang, China

^5^Ningbo Municipal Center for Disease Control and Prevention, Ningbo, China

^6^Taizhou Municipal Center for Disease Control and Prevention, Taizhou, China

^7^Department of Microbiology and Parasitology, Collaborative Innovation Center for Diagnosis and Treatment of Infectious Diseases, School of Medicine, Zhejiang University, Hangzhou, China

^8^College of Pharmaceutical Sciences, Zhejiang University of Technology, Hangzhou, China

^*^these authors contributed equally to this work

Correspondence and requests for materials should be addressed to P. P. Y. (Email:pingpingyao@aliyun.com) or H. P. Z. (Email:hanpingzhu@aliyun.com)

**
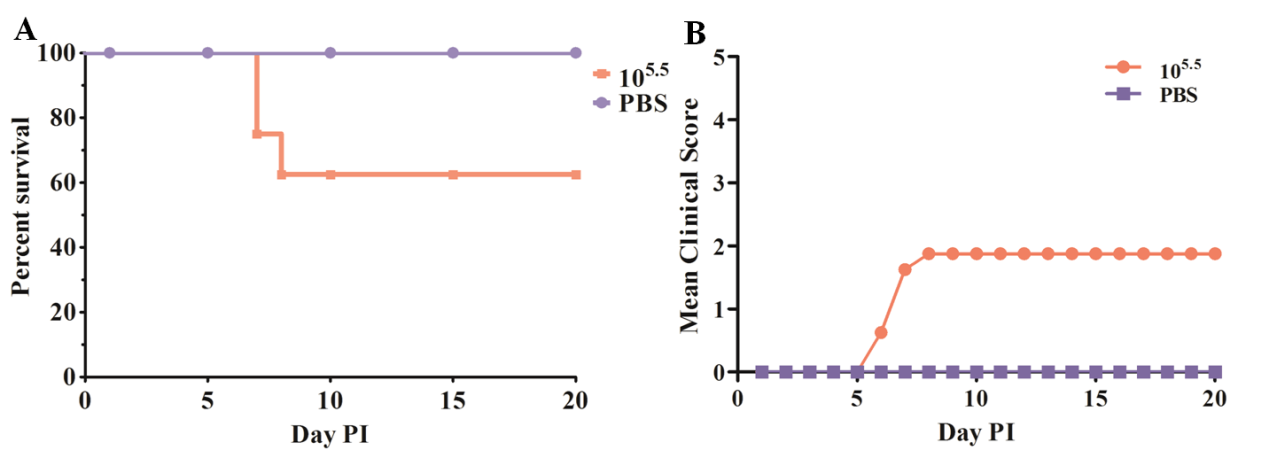
Supplementary Figure S1. Survival and severity of disease in CA16-infected gerbils by oral route.** A) Survival curves for groups of gerbils (n=8) aged 21 days when infected with CA16-194 at a TCID_50_ of 10^5.5^ or PBS by oral route. Three out of eight gerbils had disease symptoms at 6-8 dpi. B) Mean clinical scores for groups of gerbils aged 21 days when infected with CA16-194 at a TCID_50_ of 10^5.5^ or PBS by oral route. One representative of two independent experiments was shown in A and B.


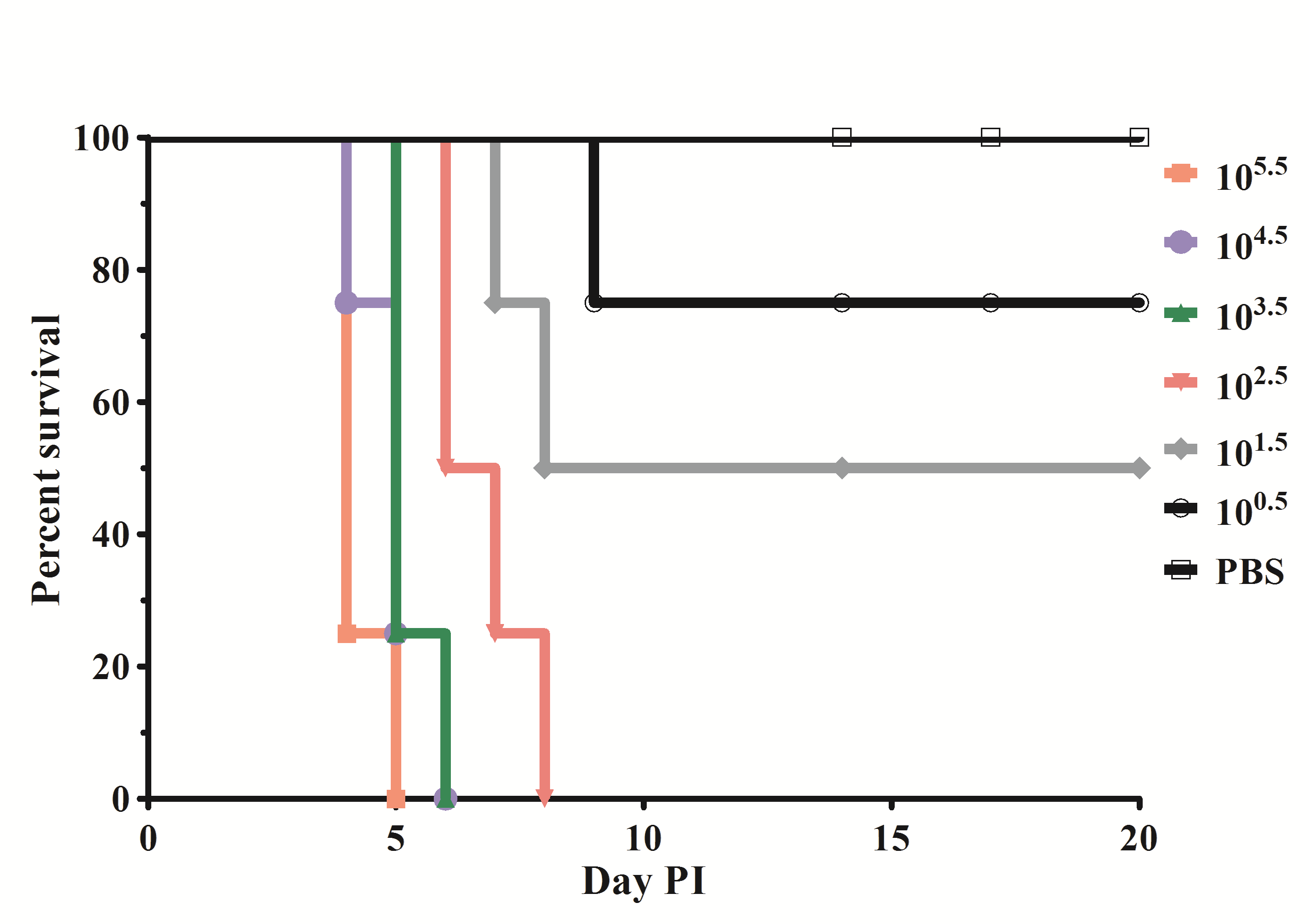


**Supplementary Figure S2. Dose-dependent survival in strain CA16-196-infected gerbils.** Survival curves for groups of gerbils (n=8) aged 21 days when infected with CA16-196 at a TCID_50_ of 10^0.5^ to 10^5.5^. The LD_50_ value of CA16-196 to gerbils were determined by Reed and Muench method and calculated as a TCID_50_ of 10^1.5^.

**
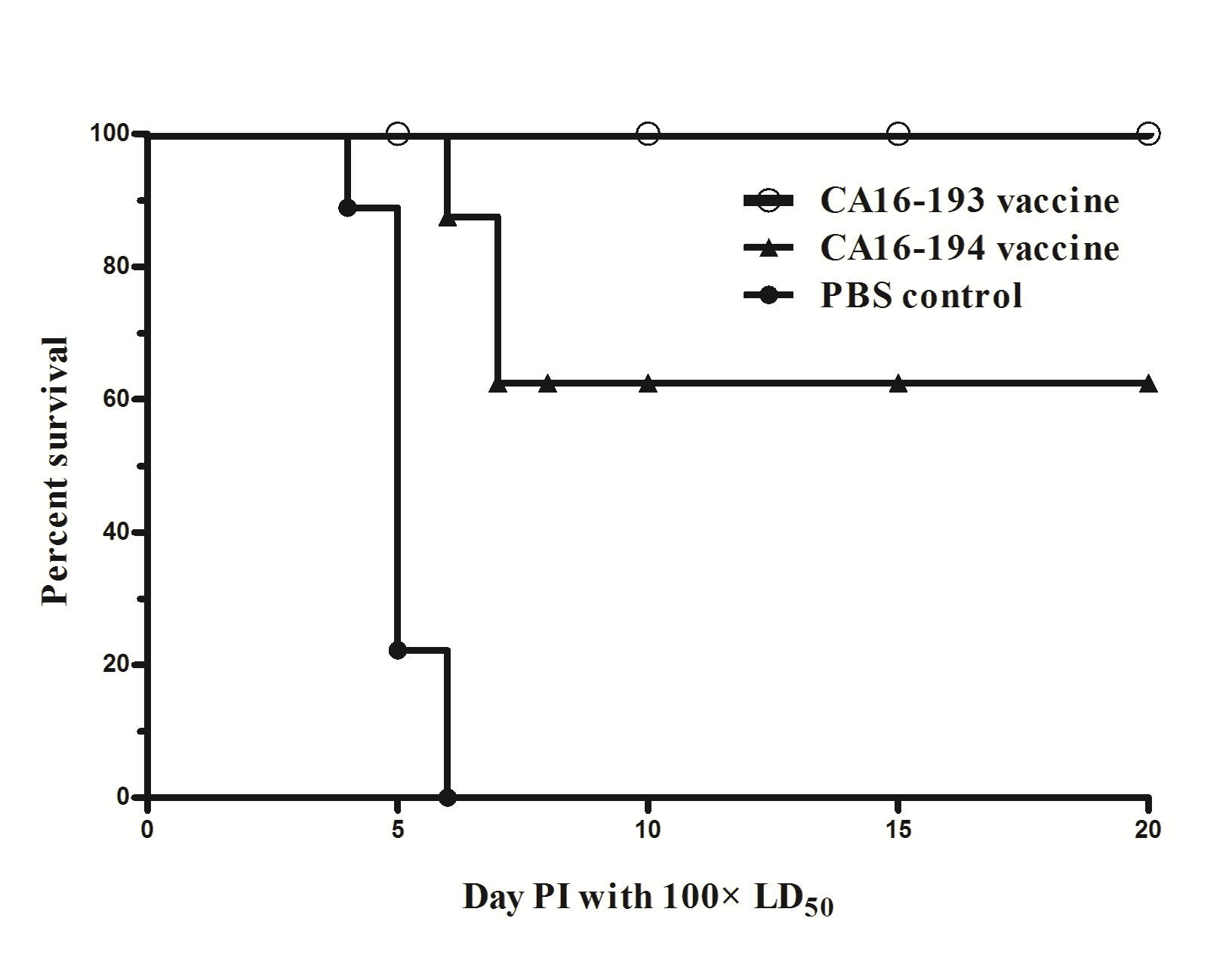
**

**Supplementary Figure S3. The antigen stability of vaccines both made from CA16-193 and CA16-194 virus.** The inactivated CA16 whole-virus vaccines were made from CA16-193 and CA16-194 virus. Both vaccines at a dose of 160 U/0.1ml were stored at 4 °C for 6 months to test the antigen stability. Gerbils were immunized with 0.1 ml CA16 vaccines or PBS as a negative control, and then challenged with CA16-196 using a 100×LD_50_. All gerbils were survival in the CA16-193 vaccine group while only 62.5% of gerbils were alive in CA16-194 vaccine group. The antigen in CA16-193 vaccine was more stable than CA16-194 vaccine.
